# Effect of pentoxifylline on preventing acute kidney injury after cardiac surgery by measuring urinary neutrophil gelatinase - associated lipocalin

**DOI:** 10.1186/1749-8090-6-8

**Published:** 2011-01-19

**Authors:** Khosro Barkhordari, Abbasali Karimi, Akbar Shafiee, Hasan Soltaninia, Mohammad Reza Khatami, Kiomars Abbasi, Fardin Yousefshahi, Babak Haghighat, Virginia Brown

**Affiliations:** 1 Department of Anesthesiology and Critical Care, Tehran Heart Center, Tehran University of Medical Sciences, Tehran, Iran; 2 Department of Cardiovascular Surgery, Tehran Heart Center, Tehran University of Medical Sciences, Tehran, Iran; 3 Leiden Academy on Vitality and Ageing, Leiden, the Netherlands; 4 Department of Nephrology, Tehran Heart Center, Tehran University of Medical Sciences, Tehran, Iran; 5 Department of Cardiothoracic Surgery and Anesthesia, Barts and the London NHS Trust, St Bartholomew's Hospital, London, UK

## Abstract

**Background:**

Based on Acute Kidney Injury Network (AKIN) criteria, we considered acute kidney injury (AKI) as an absolute increase in the serum creatinine (sCr) level of more than or equal to 0.3 mg/dl or 50%. The introduction of Urinary neutrophil gelatinase-associated lipocalin (UNGAL) has conferred earlier diagnosis of AKI. Pentoxifylline (PTX), a non-specific phosphodiesterase inhibitor, can suppress the production of some factors of inflammatory response and presumably prevent AKI. We examined the PTX on the development of AKI in cardiac surgery patients by measuring the levels of UNGAL.

**Materials and methods:**

We performed a double blind randomized clinical trial, enrolling 28 consecutive patients undergoing elective coronary artery bypass graft (CABG) surgery. Patients were divided into two groups, one to receive PTX 5 mg/kg intravenous bolus injection, followed by 1.5 mg/kg/h continuous intravenous infusion until 3 hours after cessation of CPB and the other group received placebo. UNGAL was measured before, 3 and 24 hours after surgery. In addition serum creatinine was measured before and 24, 48, 72 and 96 hours after surgery and C-reactive protein (CRP) only 24 hours postoperatively.

**Results:**

Both groups did not differ in demographic and baseline characteristics. 12 patients developed AKI 48 hours after surgery; 5 of them were in the intervention group and 7 in the control group (*p*= 0.445). There was an increase of UNGAL in both groups postoperatively, although not significant. Mean sCr was significantly increased in the control group at 24 and 48 hours after surgery (24-h mean: 0.79 ± 0.18 mg/dl vs. 1.03 ± 0.43 mg/dl, *P *value = 0.02; 48-h mean: 1.17 ± 0.24 mg/dl vs. 0.98 ± 0.20 mg/dl, *P *value = 0.03, respectively). PTX had a positive effect in preventing AKI reflecting in changes in sCr, and the increase of UNGAL was consistent with the emergence of AKI (Pearson's correlation = 0.30).

**Conclusion:**

Our study demonstrates a weak correlation between UNGAL and sCr after cardiac surgery. The rise of UNGAL in these patients may be reduced by administration of PTX although we did not show significance. PTX could reduce the occurrence of AKI as determined by attenuation of sCr rise without causing hemodynamic instability or increased bleeding. Overall, we suggest future studies with larger sample sizes to elucidate this effect and determine the different aspects of administrating PTX.

**Trial Registration:**

ISRCTN: IRCT138807302622N1

## Introduction

Acute kidney injury (AKI) is defined by the Acute kidney injury network (AKIN) as an increase in serum creatinine level (sCr) by more than 50% or 0.3 mg/dL or reduction of urine output to less than 0.5 ml/kg per hour [1]. AKI is a common and serious post operative complication and may occur in up to 50% of all patients undergoing cardiac surgery[2]. AKI is associated with 8% mortality rate compared with 0.9% in non-affected patients and remains a major factor for adverse outcomes [3].

Early measures to prevent postoperative AKI can help decreasing morbidity and mortality in these patients. Serum creatinine concentration and creatinine clearance are delayed and relatively insensitive markers of AKI. More sensitive and rapid markers of renal injury are more helpful for early diagnosis and treatment. Urinary neutrophil gelatinase-associated lipocalin (NGAL), with a molecular weight of 25 kD, is a novel biomarker that increases a few hours after a nephrotoxic or ischemic condition [3]. It has been observed that UNGAL level 2 hours after pediatric cardiac surgery can predict AKI with 100% sensitivity and 98% specificity [4].

Cardiopulmonary bypass (CPB) causes systemic inflammatory response syndrome (SIRS). Pentoxifylline (PTX), a non-specific phosphodiesterase inhibitor, inhibits some pro-inflammatory cytokines such as tumor necrosis factor (TNF)-α, interleukin -10 [5] and IL-1 [6].

Xanthine Oxidase (XO) is source of free oxygen radicals in the ischemic reperfusion injury. PTX also inhibits XO activity [7]. It can also affect the microcirculatory blood flow and contribute to the attenuation of interstitial inflammation, down regulation of monocyte chemo-attractant protein-1 gene expression, reduction in the expression of mitogenic and profibrogenic genes, and suppression of the proliferation of interstitial fibroblast and glomerulomesangial cells [8]. However, the preclinical data with regard to PTX and AKI seems controversial [9,10]. The effect of PTX in reducing renal injury by measuring sCr and α-1-microglobulin was investigated and ameasuring sCr and beneficial role of PTX on the prevention of kidney injury was observed [11]. Our study aims to investigate the effect of PTX on preventing AKI after coronary artery bypass graft (CABG) surgery, by comparing pre- and postoperative levels of NGAL and serum creatinine.

## Material and methods

After informed consent, 28 consecutive adult patients undergoing elective on-pump CABG were enrolled in doubled blind randomized control trial at Tehran Heart Center from January to June 2010. The study protocol was reviewed and approved by the Research Board the Ethics Committee at Tehran Heart Center and study funded by Tehran Heart Center. Exclusion criteria included refusal to sign the consent, Collagen-vascular disease, use of immunosuppressive agents, corticosteroids (> 3 days), methylxantines, diltiazem or sodium nitroprusside, angiography in the past 7 days, hemorrhagic diathesis and coagulopathy, uncontrolled diabetes mellitus, sepsis, renal failure (sCr > 2 mg/dl), hepatic failure (AST or ALT > 40 U/L) or urinary tract infection. The patients were randomly assigned to one of two groups: (A) the control group (placebo), and (B) the intervention group (PTX). All patients received the same anesthetic regimen and routine CPB management. Anesthesia was induced by midazolam (0.05 mg/kg), fentanyl (5 mcg/kg), propofol 2 mg/kg and pancoronium (0.1 mg/kg), and was maintained with propofol infusion (10 mg/kg/h) and additional doses of fentanyl and pancoronium. After induction of anesthesia, PTX (Aventis, Switzerland) was administered as an intravenous (IV) bolus dose of 5 mg/kg over 5 minutes, followed by 1.5 mg/kg/h slow IV infusion up to 3 hours after cessation of CPB pump. In the control group, normal saline was used as placebo. UNGAL was measured by ELISA method after induction of anesthesia and repeated at 3 and 24 hours postoperatively. Danish Bioporto^® ^rapid ELISA kits were utilized to measure UNGAL. SCr levels were measured before, 24, 48, 72 and 96 hours after surgery and C-reactive protein (CRP) checked before and 24 hours postoperatively. AKI was considered as 50% or 0.3 mg/dl increase in sCr level following surgery. Demographic characteristics, Euroscore, concurrent risk factors, infused serum volume and the time of intubation were recorded for each patient

### Statistical analysis

The results are presented as mean ± standard deviation (SD) for the quantitative variables and are summarized by absolute frequencies and percentages for categorical variables. The continuous variables were compared using Student's *t-*test or nonparametric Mann-Whitney *U *test in not normally distributed data, categorical variables were compared using *Fisher's *exact test since more than 20% of cells with expected count of less than 5 were observed. Normal distribution of data was evaluated by Kolmogorov-Smirnov test. *Pearson's *correlation coefficient was also conducted to assess the Linearity degree between the quantitative measurements. The differences between the two groups were evaluated by the two-way repeated-measures analysis of variance (ANOVA) across the all time measurements. In each ANOVA model, time was treated as a within subject factor and group and the interaction term between group and time (Group*Time) also included in the model.

For the statistical analysis, the statistical software SPSS version 13.0 for windows (SPSS Inc., Chicago, IL) was used. P values of 0.05 or less were considered statistically significant.

## Results

Half of the 28 patients undergoing CABG and met the criteria were randomized to receive PTX. Both groups were similar in demographic and pre and intraoperative characteristics. Inotropic and vasopressor drugs were used similarly in both groups during and after surgery; there was no significant difference in their doses and frequency of administration (Table 1). Number of bypass grafts in the intervention group was 3.14 ± 1.17 compared to 3.36 ± 1.75 in the control group which was not different (*p *= 0.56).

**Table 1:** Demographic and baseline data of the patients

Variable	Intervention (n = 14)	Control (n = 14)	P-value*
Male gender (n)	10 (71.4%)	13 (92.9%)	0.32

Age (year)	58.86 ± 9.41	57.14 ± 11.43	0.66

Weight (kg)	77.71 ± 10.02	82.17 ± 12.59	0.31

Euroscore (Standard score)	1.86 ± 2.07	2.14 ± 1.79	0.69

LVEF^1 ^(%)	50.00 ± 6.79	49.23 ± 7.31	0.79

Mean ABP^2 ^(mm Hg)	60.43 ± 15.32	64.86 ± 8.82	0.35

CVP (cm H_2_O)	9.00 ± 3.08	7.50 ± 2.82	0.19

Baseline sCr level (mg/dl)	0.81 ± 0.15	0.78 ± 0.18	0.56

Baseline UNGAL level (ng/ml)	10.36 ± 8.61	10.57 ± 3.54	0.93

Baseline CRP level	0.21 ± 0.12	0.32 ± 0.27	0.20

* P values <0.05 were considered as significant.

1. Left Ventricular Ejection Fraction

2. Arterial Blood Pressure

3. Central Venous Pressure

**Figure 1:**
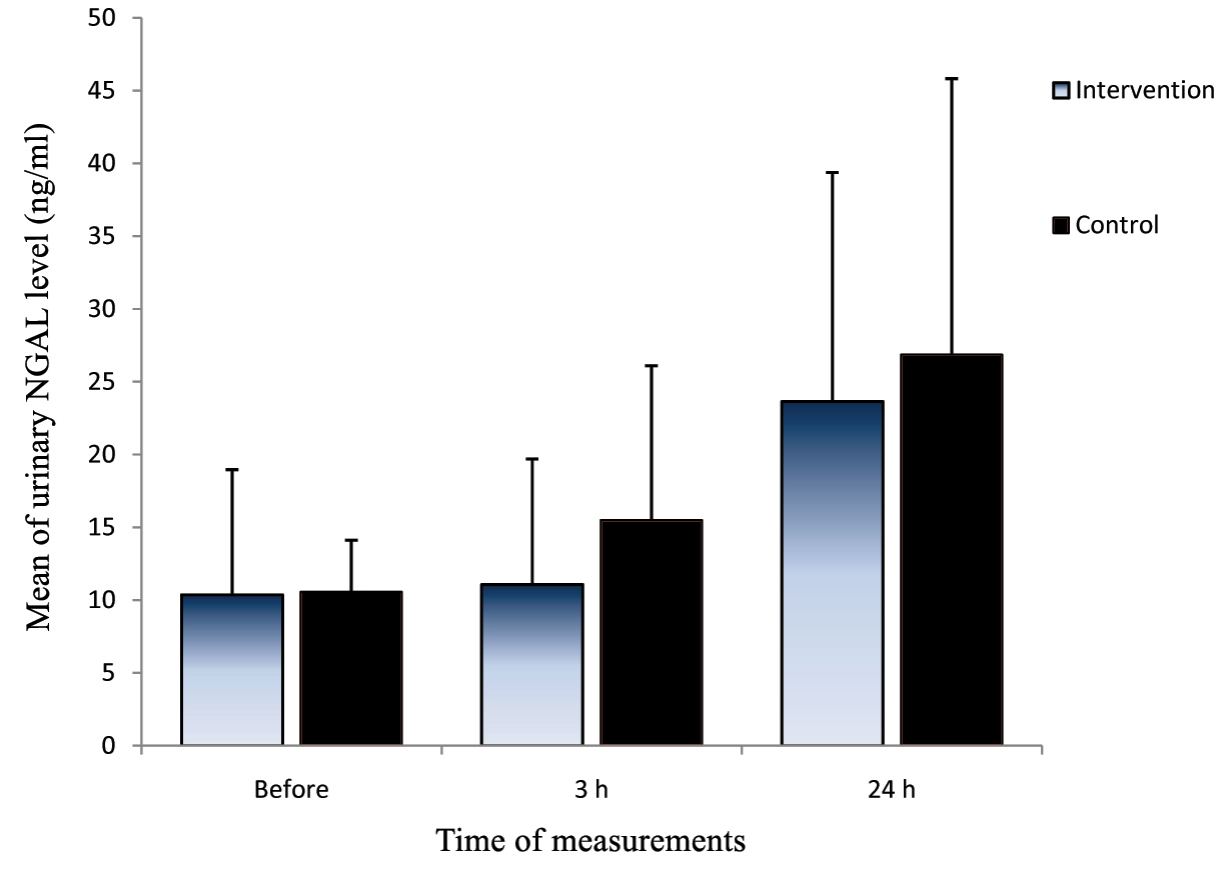
**Comparing UNGAL level before and after CABG between the intervention group and the controls (Median/interquartile range)**.

A measurement of UNGAL 3 hours after surgery showed an increase in both groups, but the difference between the groups was not significant (*p *= 0.294). Twenty four hours after surgery, the difference in rising UNGAL levels between the two groups remained insignificant (*p *= 0.587) (Figure 1). While comparing ANOVA models, the interaction terms did not reach the significant level in both models (*p *= 0.467 for Creatinine and *p *= 0.759 for NGAL).

AKI developed overall in 2 (7%) (1 in the intervention group vs. 1 in the control group) and 12(42%) of patients (5(35%) in the intervention group vs. 7(50%) in the control group) 24 and 48 hours after surgery, respectively (*p*= 0.445).

**Figure 2:**
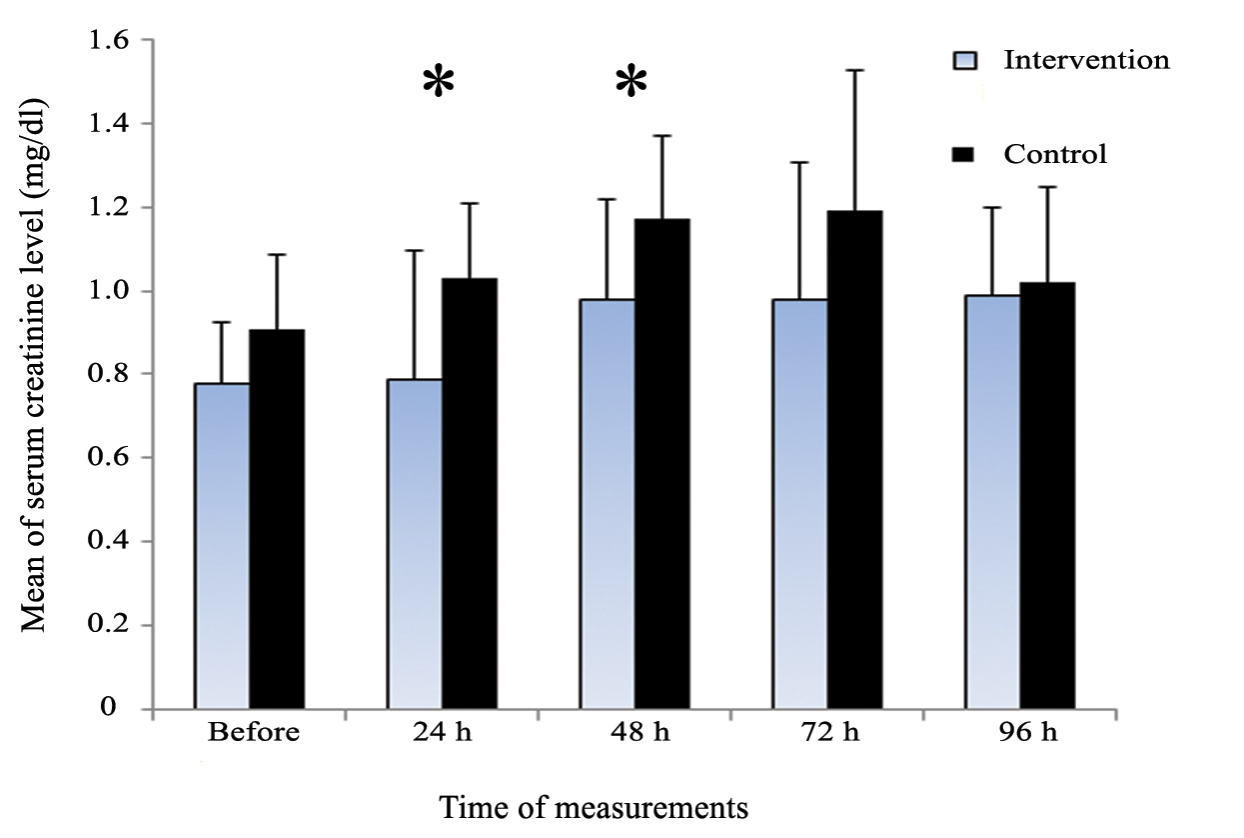
**Comparing sCr level before and after CABG between the intervention group and the controls by median/interquartile range**. Asterisks show significant difference.

There was a significant difference regarding mean sCr levels 24 and 48 hours postoperatively (*p *= 0.02 and *p *= 0.03, respectively). The level of sCr in 72 and 96 hours was not different between the groups. (*p *= 0.12 and *p *= 0.69, respectively) (Figure 2).

Overall, the increase in UNGAL was slightly correlated with the emergence of AKI as detected by sCr levels (*Pearson's *correlation coefficient r = 0.30). Also, there was no significant difference in 24 h postoperative CRP levels (*p *= 0.56) between the groups. Intubation time was also indifferent (*p *= 0.28). Common side effects of PTX (i.e., bleeding, nausea and vomiting) were not significantly higher in the intervention group than those of the control group (Table 2).

**Table 2:** Intra- and post operative variables

Variable	Intervention (n = 14)	Control (n = 14)	P-value*
***Intraoperative***			

Infused serum volume (cc)	1339.29 ± 203.97	1389.29 ± 284.32	0.59

CVP^3 ^(cm H_2_O)	9.50 ± 2.76	7.71 ± 2.12	0.67

Urine volume (cc)	1100.00 ± 427.42	1400.00 ± 514.408	0.10

Pump time (Minutes)	63.21 ± 23.69	71.50 ± 20.71	0.33

Aortic Clamp time (Min.)	37.36 ± 14.47	42.36 ± 16.14	0.39

Number of Grafts	3.14 ± 1.17	3.36 ± 0.75	0.56

Use of inotropic drugs (No. of patients)	2	5	0.32

***Postoperative***			

24-h CVP	9.68 ± 1.91	10.29 ± 3.09	0.53

24-h mean ABP	73.09 ± 21.97	80.00 ± 5.99	0.26

24-h urine volume (cc)	3864.29 ± 855.39	3693.93 ± 651.96	0.55

Bleeding (3 hours)(cc)	96.43 ± 88.71	139.29 ± 122.75	0.29

24-h bleeding (cc)	485.71 ± 200.41	500.00 ± 180.81	0.84

24-h sCr level (mg/dl)	0.79 ± 0.18	1.03 ± 0.43	0.02

48-h sCr level (mg/dl)	0.98 ± 0.20	1.17 ± 0.24	0.03

72-h sCr level (mg/dl)	1.04 ± 0.32	0.96 ± 0.24	0.46

96-h sCr level (mg/dl)	1.02 ± 0.23	0.99 ± 0.21	0.69

CRP after 24 h	11.27 ± 3.83	12.16 ± 4.18	0.56

3-h UNGAL level (ng/ml)	11.07 ± 8.63	15.50 ± 10.60	0.23

24-h UNGAL level (ng/ml)	23.64 ± 15.74	26.86 ± 18.97	0.11

Mechanical ventilation time (h)	6.91 ± 2.30	8.16 ± 3.61	0.28

Length of ICU stay (h)	43.75 ± 30.89	41.60 ± 31.46	0.63

TNG dosage (mg)	0.31 ± 0.06	0.28 ± 0.02	0.19

Heparin (IU/24 h)	26035.71	± 28714.29	± 0.14

	4343.27	4983.48	

* P values <0.05 were considered as significant.

## Discussion

Our study aimed to investigate the role of PTX in reducing AKI in patients undergoing CABG by using UNGAL. Although we could not demonstrate a significant difference between the levels of UNGAL of the control and the treatment group, surprisingly we showed a significant difference in 24 and 48 hours sCr. NGAL is a marker of injury, while creatinine is a marker of function and the expected effect would be increase of UNGAL rather than sCr. One explanation may be the size of the sample, which may be insufficient in this case.

AKI is a serious complication after cardiac surgery that occurs in 30% to 50% of all cardiac surgical patients which increases mortality and morbidity and therefore, hospital stay duration [2,3]. Patients with AKI had an eightfold increase in 30-day mortality [12].

In our study, the incidence of AKI was similar to other studies (42% after 48 hours). However, patients with a history of previous renal failure were excluded and enrolled patients had a good physiological and health condition (Euroscore 2). Thus, it seems that the real incidence of AKI among patients who undergo CABG is much higher.

Measuring urine or plasma NGAL of patients in the first hours after CPB in predicting subsequent renal injury is debatable. One study showed that measuring UNGAL level 2 hours after pediatric cardiac surgery can predict AKI with 100% sensitivity and 98% specificity. Other studies showed limited diagnostic accuracy in predicting AKI defined by change in serum creatinine after cardiac surgery [4,8,13]. A recent study on 50 patients with CPB showed a rapid incline in the postoperative UNGAL level, significantly correlated with AKI [14]. We used this biomarker as a rapid detector of AKI and surveyed the effect of PTX but could not find a strong correlation between 3 h and 12 h UNGAL measurements with 24 h and 48 h sCr levels.

Most Patients undergoing cardiac surgical procedures with CPB have SIRS [15-17] The antioxidant and anti-inflammatory effects of PTX have been showed in many studies [18,19]. PTX has also been reported to exhibit anticoagulation properties by improving red blood cell deformability, decreasing red blood cell aggregation, and inhibiting neutrophil adhesion [20].

The effect of PTX in alleviating the inflammatory process after cardiac surgery has been studied in few randomized controlled studies. Based on their results, patients receiving PTX had lower levels of inflammatory factors and reduced duration of ventilation and better outcome [21,22]. In one study, administrating PTX to elderly CABG surgical patients (age > 80) showed a significantly less rise of inflammatory factors such as PMN elastase, CRP, IL-6, IL-8 and IL-10 [21].

In another randomized clinical trial, effect of PTX on reducing the inflammatory effect following CPB surgery in 60 patients was studied by administrating either IV infusion of PTX during surgery or normal saline as placebo. Measurements of inflammatory factors (i.e., white blood cell count and differentiation, C-reactive protein, TNF-α and -6) at 6 and 24 hoursIL postoperatively showed a significant reduction in the intervention group compared to the control group[23]. In our study we could not demonstrate significant effect of PTX on CRP in the first 24 hours after surgery.

To our knowledge, this is the first published study that assesses the effect of PTX on preventing AKI by measuring UNGAL. We expected PTX to have a significant effect on reducing UNGAL regarding its anti-inflammatory and antioxidant effects in early postoperative period. However, we could not find such a correlation. This might be due to the small size of our study population, tough we must note the weak correlation of NGAL with sCr in our study.

Serum Creatinine is still a standard method for evaluating renal function. The effect of PTX in reducing renal injury by measuring sCr and a-1-microglobulin was investigated in one study and a beneficial role of PTX on prevention of kidney injury was observed [11]. Our study, confirms this effect of PTX by preventing rise of sCr at 24 and 48 hours. This may be related to other than the anti-inflammatory and antioxidant properties of PTX. We followed-up the changes in the sCr in 72 and 96 hours postoperatively and did not observe any significant change in these measurements.

We also could not find a significant effect of PTX on early outcomes such as intubation time and the duration of ICU stay as other studies claimed [22]. Again, we contribute this to small sample size.

PTX may lead to abdominal discomfort with nausea and vomiting and also has vasodilatory effect that may increase post operative bleeding [24]. There was no difference between the groups regarding homodynamic parameters or bleeding volume. This is an important finding because PTX is not desirably used in cardiac patients due to the fear from hemorrhage.

### Study limitations

The main limitation of our study is the small number of patients enrolled, which have led to a relatively low study power in finding significant differences between the two study groups. The other limitation is that the UNGAL measurement is less frequent than similar studies. We also measured CRP as an inflammatory indicator, and did not measure changes of inflammatory cytokines that may show the acute anti-inflammatory and antioxidant effects of PTX better than CRP.

## Conclusion

In this study, we show that UNGAL has a weak correlation with sCr after cardiac surgery. PTX reduces the rise of UNGAL although not significantly. PTX may have some effect on preventing AKI as determined by attenuation of creatinine rise. In our study, PTX did not increase the risk of bleeding and caused no hemodynamic instability. We suggest future larger studies to show the effect of PTX to prevent end organ damages such as AKI after cardiopulmonary bypass. More clinical trials are needed to fully determine the different aspects of administrating PTX such as standard doses, duration of infusion and its long-term effect on mortality and morbidity.

## Key messages

• Acute kidney injury (AKI) is a common complication after coronary artery bypass graft.

• Pentoxifylline tends to reduce UNGAL although not significant.

• Pentoxifylline can prevent AKI after cardiac surgery as detected by serum creatinine.

## Abbreviations

AKI: Acute Kidney Injury; AKIN: Acute Kidney Injury Network; ALT: Alanine Aminotransferase; AST: Aspartate Aminotransferase; CABG: Coronary Artery Bypass Graft; CPB: Cardiopulmonary bypass; CRP: C-reactive protein; IL: Interleukin; IV: Intravenous; PTX: Pentoxifylline; sCr: Serum Creatinine Concentration; SD: Standard Deviation; SIRS: Systemic Inflammatory Response Syndrome; TNF: Tumor Necrosis Factor; UNGAL: Urinary Neutrophil Gelatinase-Associated Lipocalin; XO: Xanthine oxidase:

## Competing interests

The authors declare that they have no competing interests.

## Authors' contributions

KB gave the conception, revised the draft and gave the final approval. AK has performed cardiac operations and acted as surgical consultant, did final scientific revision and gave final approval. AS has performed literature review, data analysis, drafting and final edition. HS has induction of Anesthesia, revised the draft and gave final approval. MK has performed as Nephrological consultant, did scientific revision and gave final approval. KA has performed cardiac operations and acted as surgical consultant, did final scientific revision and gave final approval. FY performed as Methodological consultant, did scientific revision and gave final approval. BH performed as Methodological consultant, did scientific revision and gave final approval. VB did the final language edit and gave final approval.

## References

[B1] Mehta RL Kellum JA Shah SV Molitoris BA Ronco C Warnock DG Levin A Acute Kidney Injury Network: Report of an initiative to improve outcomes in acute kidney injury Crit Care 2007 11 R31 10.1186/cc5713 17331245 PMC2206446

[B2] Haase M Haase-Fielitz A Bagshaw SM Ronco C Bellomo R Cardiopulmonary bypass-associated acute kidney injury: a pigment nephropathy? Contrib Nephrol 2007 156 340 353 full_text 17464145 10.1159/000102125

[B3] Rosner MH Okusa MD Acute kidney injury associated with cardiac surgery Clin J Am Soc Nephrol 2006 1 19 32 10.2215/CJN.00240605 17699187

[B4] Mishra J Dent C Tarabishi R Mitsnefes MM Ma Q Kelly C Ruff SM Zahedi K Shao M Bean J Mori K Barasch J Devarajan P Neutrophil gelatinase-associated lipocalin (NGAL) as a biomarker for acute renal injury after cardiac surgery Lancet 2005 365 1231 8 10.1016/S0140-6736(05)74811-X 15811456

[B5] Visser J Groen H Klatter F Rozing J Timing of pentoxifylline treatment determines its protective effect on diabetes development in the Bio Breeding rat Eur J Pharmacol 2002 445 133 40 10.1016/S0014-2999(02)01625-4 12065204

[B6] Sullivan GW Carper HT Novick WJ Jr Mandell GL Inhibition of the inflammatory action of interleukin-1 and tumor necrosis factor (alpha) on neutrophil function by pentoxifylline Infect Immun 1988 56 1722 9 2838424 10.1128/iai.56.7.1722-1729.1988 PMC259468

[B7] Hammerman C Goldschmidt D Caplan MS Kaplan M Schimmel MS Eidelman AI Branski D Hochman A Amelioration of ischemia-reperfusion injury in rat intestine by pentoxifylline-mediated inhibition of xanthine oxidase J Pediatr Gastroenterol Nutr 1999 29 69 74 10.1097/00005176-199907000-00017 10400107

[B8] Wu HM Yuan QY Zhou RL Li J Liu GJ Pentoxifylline for diabetic kidney disease (Protocol) Cochrane Database of Systematic Reviews 2007 4 CD006800 10.1002/14651858.CD006800.pub2 PMC11831249 22336824

[B9] Okumura AS Rodrigues LE Martinelli R Pentoxifylline in ischemia-induced acute kidney injury in rats Ren Fail 2009 31 829 32 10.3109/08860220903137509 19925292

[B10] Groesdonk HV Bauer A Kreft B Heringlake M Paarmann H Pagel H Urodilatin and pentoxifylline prevent the early onset of Escherichia coli-induced acute renal failure in a model of isolated perfused rat kidney Kidney Blood Press Res 2009 32 81 90 10.1159/000209378 19321979

[B11] Boldt J Brosch C Piper SN Suttner S Lehmann A Werling C Influence of prophylactic use of pentoxifylline on postoperative organ function in elderly cardiac surgery patients Crit Care Med 2001 29 952 8 10.1097/00003246-200105000-00008 11378603

[B12] Kheterpal S Tremper KK Heung M Rosenberg AL Englesbe M Shanks AM Campbell DA Development and validation of an acute kidney injury risk index for patients undergoing general surgery: results from a national data set Anesthesiology 2009 110 505 15 10.1097/ALN.0b013e3181979440 19212261

[B13] Wagener G Gubitosa G Wang S Borregaard N Kim M Lee HT Urinary Neutrophil Gelatinase-Associated Lipocalin and Acute Kidney Injury After Cardiac Surgery Am J Kidney Dis 2008 52 425 33 10.1053/j.ajkd.2008.05.018 18649981

[B14] Tuladhar SM Püntmann VO Soni M Punjabi PP Bogle RG Rapid detection of acute kidney injury by plasma and urinary neutrophil gelatinase-associated lipocalin after cardiopulmonary bypass J Cardiovasc Pharmacol 2009 53 261 6 10.1097/FJC.0b013e31819d6139 19247188

[B15] Asimakopoulos G Gourlay T A review of anti-inflammatory strategies in cardiac surgery Perfusion 2003 18 7 12 10.1191/0267659103pf623oa 12708760

[B16] Chong AJ Hampton CR Verrier ED Microvascular Inflammatory Response in Cardiac Surgery Seminars in Cardiothoracic and Vascular Anesthesia 2003 7 333 54

[B17] Laffey JG Boylan JF Cheng DC The systemic inflammatory response to cardiac surgery: implications for the anesthesiologist Anesthesiology 2002 97 215 52 10.1097/00000542-200207000-00030 12131125

[B18] Radfar M Larijani B Hadjibabaie M Rajabipour B Mojtahedi A Abdollahi M Effects of pentoxifylline on oxidative stress and levels of EGF and NO in blood of diabetic type-2 patients; a randomized, double-blind placebo-controlled clinical trial Biomed Pharmacother 2005 59 302 6 10.1016/j.biopha.2005.05.003 15932791

[B19] Zhang M Xu YJ Saini HK Turan B Liu PP Dhalla NS Pentoxifylline attenuates cardiac dysfunction and reduces TNF-alpha level in ischemic-reperfused heart Am J Physiol Heart Circ Physio 2005 289 H832 9 10.1152/ajpheart.00178.2005 15833806

[B20] Chapelier A Reignier J Mazmanian M Detruit H Dartevelle P Parquin F Cerrina J Le Roy Ladurie F Hervé P Pentoxifylline and lung ischemia-reperfusion injury: application to lung transplantation. Université Paris-Sud Lung Transplant Group J Cardiovasc Pharmacol 1995 25 Suppl 2 S130 3 10.1097/00005344-199500252-00027 8699852

[B21] Boldt J Brosch C Lehmann A Haisch G Lang J Isgro F Prophylactic use of pentoxifylline on inflammation in elderly cardiac surgery patients Ann Thorac Surg 2001 71 1524 9 10.1016/S0003-4975(01)02462-6 11383794

[B22] Heinze H Rosemann C Weber C Heinrichs G Bahlmann L Misfeld M Heringlake M Eichler W A single prophylactic dose of pentoxifylline reduces high dependency unit time in cardiac surgery - a prospective randomized and controlled study Eur J Cardiothorac Surg 2007 32 83 9 10.1016/j.ejcts.2007.04.011 17499999

[B23] Cağ li K Ulaş MM Ozişik K Kale A Bakuy V Emir M Balci M Topbaş M Sener E Taşdemir O The intraoperative effect of pentoxifylline on the inflammatory process and leukocytes in cardiac surgery patients undergoing cardiopulmonary bypass Perfusion 2005 20 45 51 15751670 10.1191/0267659105pf779oa

[B24] Hemmer CJ Hort G Chiwakata CB Seitz R Egbring R Gaus W Hogel J Hassemer M Nawroth PP Kern P Dietrich M Supportive pentoxifylline in falciparum malaria: no effect on tumor necrosis factor alpha levels or clinical outcome: a prospective, randomized, placebo-controlled study Am J Trop Med Hyg 1997 56 397 403 9158047 10.4269/ajtmh.1997.56.397

